# Lost in Translation: Defects in Transfer RNA Modifications and Neurological Disorders

**DOI:** 10.3389/fnmol.2017.00135

**Published:** 2017-05-09

**Authors:** Andrea Bednářová, Marley Hanna, Isabella Durham, Tara VanCleave, Alexis England, Anathbandhu Chaudhuri, Natraj Krishnan

**Affiliations:** ^1^Department of Biochemistry and Physiology, Institute of Entomology, Biology Centre, Academy of SciencesČeské Budějovice, Czechia; ^2^Laboratory of Molecular Biology and Biochemistry, Department of Biochemistry, Molecular Biology, Entomology and Plant Pathology, Mississippi State UniversityMississippi State, MS, USA; ^3^Molecular Biosciences Program, Arkansas State UniversityJonesboro, AR, USA; ^4^Department of Wildlife, Fisheries and Aquaculture, Mississippi State UniversityMississippi State, MS, USA; ^5^Department of Natural Sciences, Stillman CollegeTuscaloosa, AL, USA

**Keywords:** transfer RNA modifications, modified nucleosides, neurological disease, queuosine, Q-tRNA

## Abstract

Transfer RNAs (tRNAs) are key molecules participating in protein synthesis. To augment their functionality they undergo extensive post-transcriptional modifications and, as such, are subject to regulation at multiple levels including transcription, transcript processing, localization and ribonucleoside base modification. Post-transcriptional enzyme-catalyzed modification of tRNA occurs at a number of base and sugar positions and influences specific anticodon–codon interactions and regulates translation, its efficiency and fidelity. This phenomenon of nucleoside modification is most remarkable and results in a rich structural diversity of tRNA of which over 100 modified nucleosides have been characterized. Most often these hypermodified nucleosides are found in the wobble position of tRNAs, where they play a direct role in codon recognition as well as in maintaining translational efficiency and fidelity, etc. Several recent studies have pointed to a link between defects in tRNA modifications and human diseases including neurological disorders. Therefore, defects in tRNA modifications in humans need intensive characterization at the enzymatic and mechanistic level in order to pave the way to understand how lack of such modifications are associated with neurological disorders with the ultimate goal of gaining insights into therapeutic interventions.

Chemical modifications of RNA is a ubiquitous phenomenon in all kingdoms of life (Grosjean, 2009) and more than 100 chemically distinct modifications in cellular RNA have been identified to date. Initially, studies on such modifications were restricted to the ones that were the most abundant such as: peudouridine (ψ), N^1^-methyladenosine (m^1^A) and 2′-O-methylation (2′OMe) in ribosomal RNA (rRNA) and transfer RNA (tRNA), and 2′OMe, N^6^-methyladenosine (m^6^A) in messenger RNA (mRNA) and viral RNA (Desrosiers et al., 1974; Perry and Kelley, 1974; Dubin and Taylor, 1975). With technological advances in high throughput sequencing and mass spectrometry, additional less prevalent modifications on less abundant RNA species are being discovered (Nachtergaele and He, 2017; Song and Yi, 2017) and their biological significance uncovered.

## tRNA Modifications

tRNA molecules translate the genetic code by recognizing cognate mRNA codons during the process of translation and protein synthesis. This ribosome-mediated interaction of the mRNA codons with the anticodon of the tRNA results in the discrimination of cognate vs. near-cognate and non-cognate codons (Ogle et al., 2001; Yusupov et al., 2001). The tRNAs undergo extensive post-transcriptional modifications during the process of maturation. In eukaryotes, around 100 different chemical modifications have been described occurring at different positions on the tRNA (Jackman and Alfonzo, 2013; tRNA modification database^1^ and MODOMICS^2^). Among all the modified nucleosides observed in tRNA, eight are present at the same position and the same subpopulation of tRNA isoacceptors in all domains of life, suggesting that tRNA modifications have highly conserved origins and functions (Björk, 1986; Björk et al., 2001). The largest diversity of the post-transcriptional nucleoside modifications occurs at the wobble position 34 in the anticodon or immediately 3′ adjacent to the anticodon triplet at position 37 (Rozenski et al., 1999; Dunin-Horkawicz et al., 2006).

## Elusive Biological Functions of tRNA Modifications

A major limitation in the study of the biological significance and function of tRNA base modifications has been the lack of a phenotype associated with such modifications under defined settings. However, with technological advances, several modification defects and their phenotypes have now been described which provides insights into their biological roles (Hopper and Phizicky, 2003). In general, the modifications of bases at the wobble position in the anticodon or immediately adjacent to the anticodon triplet often impact the decoding abilities of tRNA’s by restriction and/or improvement of the codon-anticodon interactions, which may affect maintenance of the reading frame (Agris, 1991; Lim, 1994; Björk, 1995; Yokoyama and Nishimura, 1995; Johansson et al., 2008). Some modified bases present in the anticodon loop may be required for aminoacylation (Giege et al., 1998), while other modifications outside the anticodon loop, e.g., m^1^A_58_ of tRNA_i_^Met^, may be important for the structure or stability of the tRNA (Anderson et al., 1998; Calvo et al., 1999; Kadaba et al., 2004). Some of these tRNA base modifications may also regulate the speed and fidelity of translation (Hori, 2014; Manickam et al., 2016). There is also evidence that the loss of certain single modifications can be compensated by the presence of others, which suggests the existence of some redundancy in the system (Alexandrov et al., 2006). This indicates that modifications in tRNA affect translational function in very subtle ways and their main goal is to maintain tRNA functionality in the cell.

The cell utilizes the tRNA nucleoside modifications which are located in or near the anticodon loop to alter the repertoire of proteins that are preferentially synthesized. The affinity of codon-anticodon interaction can also regulate the biased subsets of mRNA, and so by adjusting the frequency of modifications in tRNA, the cells are capable of rapidly reacting to environmental challenges and thus focus their synthesis capacity on the proteins needed the most during these stress situations. For example, in *Escherichia coli*, stress resistance was significantly altered when heat shock genes were targeted by closely associated mutations (Krisko et al., 2014).

A feature of wobble modifications is their frequent tRNA specificity. It has been shown in yeast (*Saccharomyces cerevisiae*) that tRNA methyltransferase 4 (Trm4), which is multi-site specific, catalyzes 5-methylcytosine (m^5^C) formation in over 34 species of tRNA and this occurs at position 48 (between variable arm and T stem loop) most frequently (Czerwoniec et al., 2009). However, the only tRNA with m^5^C at the wobble position is tRNA^Leu(CAA)^ and this uniqueness has a role in the regulation of translation in response to oxidative stress in cells (Chan et al., 2012; Gu et al., 2014). This has given rise to the concept of tRNA modification tunable transcripts (MoTTs; Endres et al., 2015). This concept is defined as tRNA modifications that lead to: (a) transcripts that use specific degenerate codons and codon biases to encode critical stress response proteins; and (b) transcripts whose translation is influenced by changes in wobble base tRNA modification.

## Defects in tRNA Modifications Associated with Neurological Disorders

Although the role of tRNA in the process of translation has been known since the late 1950s it is only in 1990, a mutation in tRNA was linked to a human disease (Kobayashi et al., 1990). The range of disorders now associated with defects in tRNA modifications range from metabolic (Type II Diabetes), respiratory defects and myopathies, mitochondrial disorders such as mitochondrial myopathy, encephalopathy, lactic acidosis and stroke-like episodes (MELAS) and myoclonus epilepsy associated with ragged-red fibers (MERRF), to X-linked intellectual disability and familial dysautonomia (Duechler et al., 2016).

In *S. cerevisiae*, many of the tRNA modifications and the enzymes involved have been well characterized (Phizicky and Hopper, 2010). However, the homologs for many of these enzymes and their biological significance in humans have only recently started to emerge (Towns and Begley, 2012). This has led to a new appreciation of the link between tRNA modifications and human diseases (Torres et al., 2014; Hou et al., 2015).

**Figure 1:**
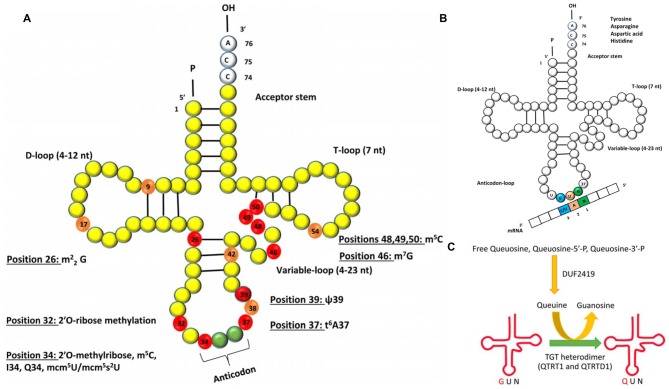
**(A)** Schematic representation of the secondary structure of transfer RNA (tRNA) with post-transcriptionally modified residues in light orange and red (numbered). The residues marked in red are of relevance to human neurological disorders. The abbreviations for the modifications are as follows: m^2^_2_G: N2, N2-dimethyl guanosine; m^5^C: 5-methylcytosine; I34: inosine at position 34; Q34: queuosine at position 34, mcm^5^U: 5-methoxycarbonylmethyluridine; mcm^5^s^2^U: 5-methoxycarbonylmethyl-2-thiouridine; ψ: pseudouridine; t^6^A37: N^6^-threonyl-carbamoyl-adenosine at position 37. **(B)** Queuosine modification of tRNA. The G_34_U_35_N_36_ anticodon sequence of tRNA isoacceptors for amino acids tyrosine, asparagine, aspartic acid and histidine will base pair with a N_1_A_2_C/U_3_ codon of mRNA. G = guanine, U = uridine, A = adenine, N = any base. **(C)** Single step of Q modification of tRNAs in eukaryotes showing replacement of G_34_ by Q in the anticodon triplet by the enzyme heterodimeric TGT enzyme complex. Free Queuosine, Queuosine-5′-P, Queuosine-3′-P are obtained from degradation of Q-tRNA. Salvage of Q is accomplished by protein DUF2419.

The human brain is particularly sensitive to defects in tRNA modifications and several neurological disorders can be attributed to mutations in genes that affect the post-transcriptional modifications that occur in certain residues of the tRNA (Figure 1A; Table 1).

**Table 1:** **Transfer RNA (tRNA) modifications, genes affected and their associated neurological disorders**.

tRNA modification and residues affected	Gene(s) affected	Neurological disorders	References
m^2^_2_G, several tRNA’s, 26	*TRMT1*	Cognitive dysfunction/Intellectual disability	Najmabadi et al. (2011)
2′-O-methylribose Cm, Gm, ncm^5^Um (tRNA)^Leu, Trp, Phe^, 32 and 34	*FTSJ1*	X-linked mental retardation	Gong et al. (2008), Guy et al. (2015)
m^5^C, tRNA^Leu^, 34 Several tRNA’s 48, 49, 50	*NSUN2*	Autosomal-recessive intellectual disability	Abbasi-Moheb et al. (2012), Ghadami et al. (2015)
		Dubowitz-like syndrome	Martinez et al. (2012)
U_39_ to ψ, tRNA^Phe^, 39	*PUS3*	Cognitive dysfunction/Intellectual disability	Shaheen et al. (2016)
A to I editing (tRNA)^Ala, Pro, Thr, Val, Ser, Arg, Leu, Ile^, 34	*ADAT3*	Intellectual disability	Alazami et al. (2013)
mcm^5^s^2^U, ncm^5^U, and derivatives, 34	*IKBKAP, ELP2, ELP3, ELP4*	Intellectual disability	Najmabadi et al. (2011), Cohen et al. (2015)
		Familial dysautonomia	Karlsborn et al. (2014)
		Amyotrophic lateral sclerosis	Simpson et al. (2009)
		Rolandic epilepsy	Reinthaler et al. (2014)
G to Q, (tRNA)^Tyr, Asn, Asp, His^, 34	*TGT*	Encephalomyelitis	Varghese et al. (2017)
m^7^G, several tRNA’s, 46	*WDR4*	Downs syndrome	Michaud et al. (2000)

## Cognitive Disorders and Intellectual Disability

Dimethylation of guanosines (m^2^_2_G) occurs at position 26 of tRNAs and this is catalyzed by the human tRNA methyltransferase 1 (Liu and Straby, 2000; Figure 1A). Inactivation of this gene by a homozygous frameshift mutation is a biomarker for recessive cognitive disorders (Najmabadi et al., 2011). In human tRNAs so far characterized, pseudouridine is located at 13 different positions and different pseudouridylases (Pus) catalyze the modification at each of the site(s). It was recently reported that a nonsense mutation in *PUS3* gene known for its role in isomerizing uracil to pseudouridine via Pus3 at position 39 in human tRNA was significantly reduced in patients with intellectual disability (Shaheen et al., 2016, Table 1).

Positions 32 and 34 on tRNA^Leu^, tRNA^Trp^ and tRNA^Phe^ are methylated (Figure 1A) by the gene encoding a methyltransferase—the FtsJ methyltransferase homolog 1 (*FTSJ1*), homologous to the yeast methyltransferase 7 (TRM7). Non-syndromic X linked mental retardation and intellectual disability are associated with mutations in this gene (Gong et al., 2008; Guy et al., 2015). At position 34 of tRNALeu and also at positions 48–50 on several tRNAs, m^5^C formation occurs (Brzezicha et al., 2006; Hussain et al., 2013; Khoddami and Cairns, 2013; Figure 1A). In higher eukaryotes, the only known m^5^C RNA methyltransferases are NSun2 and Dnmt2 and both enzymes are confirmed to target tRNA (Brzezicha et al., 2006). Deficiency in Nsun2 has been linked to intellectual disability (Abbasi-Moheb et al., 2012; Ghadami et al., 2015; Table 1). Interestingly, deletion of the ortholog of Nsun2 in fly results in severe short-term memory deficits (Abbasi-Moheb et al., 2012). Dubowitz-like syndrome associated with intellectual disability is also linked to mutations in Nsun2 (Martinez et al., 2012). Adenosine deaminase catalyze the conversion of adenosine-to-inosine at position 34 (I34) of tRNAs (Figure 1), and is encoded by heterodimeric adenosine deaminase (hetADAT). A single missense mutation in *ADAT3* encoding for one of the subunits of hetADAT is present in families with individuals affected with intellectual disability (Alazami et al., 2013). 7-methylguanosine (m^7^G) modification of tRNA occuring in eukaryotes and bacteria, is nearly always found at position 46 (Figure 1), and is a modification that confers a positive charge to the base. The human WD repeat domain 4 (WDR4) is the closest homolog to the yeast TRM82 protein complex responsible for formation of m7G. WDR4 has been identified in a search for candidate genes of Down’s syndrome phenotypes (Michaud et al., 2000), but a direct association has not yet been demonstrated.

## Neurodevelopmental Disabilities

Elongator is a highly conserved multi-subunit protein complex (ELP 1–6) that is essential to transcription elongation, histone acetylation as well as tRNA modification. The association between elongator complex and translational fidelity via regulation of tRNA modifications has gathered accumulating evidence in the last decade (Kojic and Wainwright, 2016). In eukaryotes, uridine at position 34 are modified to 5-carbamoyl-methyl-uridine (ncm^5^U), 5-methoxy-carbonyl-methyl-uridine (mcm^5^U), or 5-methoxy-carbonyl-methyl-2-thio-uridine (mcm^5^2U) in the anticodons of tRNA^Lys^, tRNA^Glu^ and tRNA^Gln^. These modifications require the elongator complex (Esberg et al., 2006; Johansson et al., 2008; Bauer and Hermand, 2012). The Elongator mediated transfer of methyl-group to tRNA U_34_ involves a SAM-mediated mechanism coupled with an electron transfer from Kti11/Kti13, a cofactor complex (Boal et al., 2011; Kolaj-Robin et al., 2015). Recently, Glatt et al. (2016) showed the structural basis for such a modification of tRNA by ELP3. Moreover, it has been shown that a missense variant of the gene encoding for ELP2 is linked to neurodevelopmental disabilities (Najmabadi et al., 2011; Cohen et al., 2015).

## Rolandic Epilepsy

Rare and deleterious variants of ELP4 have been associated with atypical rolandic epilepsy (RE; Reinthaler et al., 2014, Table 1). ELP4 has a putative role in neuronal migration and variants in the gene confer susceptibility to parts of the RE disease spectrum.

## Motor Neuron Diseases

Allelic variants of ELP3 were associated with amyotrophic lateral sclerosis (ALS; a spontaneous, progressive motor neuron disease), in three human populations (Simpson et al., 2009), and, in the same study, two different loss of function mutations in ELP3 and genes important for neuronal communication and survival were identified in a mutagenesis screen in *Drosophila*. Moreover, dose-dependent motor axonal abnormalities were observed in zebrafish embryos when ELP3 protein levels were knocked down using antisense morpholinos. (Simpson et al., 2009). Mutations in ELPC1 and ELPC3 in *Caenorhabditis elegans* are associated with neurological and developmental dysfunctions (Chen et al., 2009). These studies implicate the importance of the elongator complex which are essential for tRNA modifications.

Interestingly, phenotypes associated with defective Elongator are due to lack of formation of mcm^5^s^2^U at position 34 of tRNAs (Phizicky and Hopper, 2010; Figure 1A). Mutation in the inhibitor of kappa light polypeptide gene enhancer in B-cells, kinase complex-associated protein (*IKBKAP*) gene leads to reduced levels of the modified nucleoside mcm^5^s^2^U in tRNA and is associated with familial dysautonomia, a recessive neurodegenerative genetic disease (Karlsborn et al., 2014). Since Elongator influences acetylation of α-tubulin in neurons, there is a strong possibility that it may also play a significant role in neurological disorders such as Huntington’s disease, Alzheimer’s disease, Parkinson’s disease and ALS (Nguyen et al., 2010).

## Neurodegeneration

The N^6^-threonyl-carbamoyl-adenosine (t^6^A) modification, is a complex modification of adenosine located at position 37 (t^6^A37) next to the anticodon stem loop of many tRNA’s that decode the ANN codons (Figure 1A). A recent report on a biosynthetic defect of the t^6^A molecule as a result of a mutation to kinase-associated endopeptidase (*KAE1*) gene of the highly conserved Kinase, Endopeptidase and Other Proteins of small Size (KEOPS) complex manifested in a neurodegenerative phenotype in two patients (Edvardson et al., 2017). This adds to the growing list of defects in cytoplasmic tRNA modification enzymes associated with neurological disorders.

## Significance of Queuosine Modification of Trna and Its Role in Multiple Sclerosis and Other Neurological Disorders

Queuosine (Q) is among the most elaborate of the known tRNA modifications occurring in the wobble base (position 34) of tRNAs with G_34_U_35_N_36_ anticodons (tRNA_GUN_; where *N* = any base) that incorporate tyrosine, asparagine, aspartic acid or histidine amino acids (Katze et al., 1982; Nishimura, 1983; El Yacoubi et al., 2012; Figure 1B). This modification is widely distributed in most prokaryotic and eukaryotic phyla with the exception of yeast and mycoplasma (Katze et al., 1982). Structurally, Q comprises a 7-deazaguanosine core (Iwata-Reuyl, 2003). Q base is known to exist in four forms in biological systems: free nucleoside, free nucleotide and nucleoside incorporated into tRNA and free queuine base. tRNA-guanine transglycosylase (TGTase; EC 2.4.2.29) which was renamed later as tRNA-guanine ribosyltransferase (TGRase) by the Enzyme Commission, is the enzyme that catalyzes the formation of this modified tRNA (Farkas et al., 1984). Even though the Q modification is widely distributed in Bacteria and Eukarya, it is synthesized *de novo* by most bacteria, whereas all eukaryotes solely rely on salvage from environment to incorporate this complex modification. In eukaryotes, queuosine production is from the fully formed queuine base obtained from diet or microflora in intestinal, circulatory or membrane transport systems and incorporated post-transcriptionally by the eukaryotic analog of the prokaryotic TGTase. The completely modified base queuine is utilized by eukaryotic TGTase as a substrate and is irreversibly incorporated by a base-exchange reaction at position 34 of specific tRNAs (Kersten and Kersten, 1990; Figure 1C). Eukaryotic TGTase was shown to be a heterodimeric complex of 100–104 kDa protein, comprising a putative 60–66 kDa (QTRTD1) regulatory subunit and a 34–45 kDa (QTRT1) catalytic subunit (Morris et al., 1995; Slany and Müller, 1995; Deshpande et al., 1996). The former is probably involved in tRNA binding (Chen et al., 2010). The identification of DUF2419 as a potential member of the Q salvage protein family involved in Q-recycling was recently reported from phylogenetic analysis of plant, animal and fungal micronutrient Q salvage systems (Zallot et al., 2014). Queuosine modification system in mammalian cells is dependent on: (a) the uptake of queuine base into the cells by a queuine-specific membrane transport system (Elliott and Crane, 1990; Morris et al., 1996, 1999); (b) enzymatic incorporation of queuine into the first position of the anticodon loop of tRNA by TGTase (Morris et al., 1995, 1996, 1999; Slany and Müller, 1995); and (c) salvage of queuine by DUF2419 from queuosine 5′ monophosphate, free queuosine or queuosine-3′ monophosphate, resulting from tRNA degradation (Gündüz and Katze, 1982, 1984; Morris et al., 1999; Zallot et al., 2014).

The physiological significance of Q-tRNA and role of queuine has not been comprehensively established in eukaryotes. The absence of Q leads to no obvious developmental phenotypes in *C. elegans* (Gaur et al., 2007), in contrast, Q-deficient *Drosophila* are more sensitive to cadmium stresses (Siard et al., 1991). In mammals, the absence of both Q and tyrosine cause severe symptoms leading to death (Marks and Farkas, 1997). This suggests that queuine, the Q precursor is an essential micronutrient and also plays an important role in biosynthesis of Q to form Q-tRNA (Nishimura, 1983; Kersten, 1988). Queuine has been shown to promote the activity of antioxidant enzymes and thus may have a role in improving the antioxidant defense system (Pathak et al., 2008). Queuine has been shown to induce cell differentiation in cancerous cells (Chen and Wu, 1994). Lack of Q in first position of anticodons in tRNAs of Q-family is reported in various tumor cells (Dirheimer et al., 1995). The histopathological grade of malignancy is strongly correlated to amount of Q-deficient tRNA, and increased deficiency is observed in metastatic ovarian malignant tumors compared with primary malignancies (Baranowski et al., 1994). In short, Q modification of tRNA has been shown to play a divergent role in cellular machinery (Vinayak and Pathak, 2010).

Autoimmune diseases, including multiple sclerosis, are characterized by the rapid expansion of T cells directed to self-antigens. The potential medicinal relevance of targeting the hypomodification of Q-tRNA was recently demonstrated in the treatment of a chronic multiple sclerosis model—murine experimental autoimmune encephalomyelitis (Varghese et al., 2017). The administration of a *de novo* designed eukaryotic TGT substrate (NPPDAG) led to an unprecedented complete reversal of clinical symptoms and a dramatic reduction of markers associated with immune hyperactivation and neuronal damage. Thus, TGT is essential for the therapeutic effect, since animals deficient in TGT activity were refractory to therapy. The data suggests that exploitation of the eukaryotic TGT enzyme is a promising approach for the treatment of multiple sclerosis.

It has also been shown that deficiency in queuine in human HepG2 cells and mice made deficient in Q-tRNA, by disrupting the tRNA TGTase, have impaired ability to produce tyrosine from phenylalanine. This is because of a decrease in supply of the cofactor BH_4_ by increased oxidation of tetrahydrobiopterin leading to elevation of dihydrobiopterin (BH_2_) by an as yet undetermined mechanism (Rakovich et al., 2011). This has significant implications for dopamine (DA) biosynthesis which depends on a robust supply of BH_4_ (Nagatsu and Ichinose, 1996). It is thus interesting to speculate if such a deficiency of Q-tRNA could mimic Parkinsonian disorders.

All these defects in tRNA modifications and the consequent neurological disorders strongly indicate a functional association between aberrant tRNA modification and development of neurological disease. This raises a question of how many more defective proteins lead to lack of tRNA modifications and could these also lead to human pathologies, particularly neurological and/or neurodegenerative disorders?

## Conclusions

Complex clinical pathologies arise as a result of mutations in tRNA genes and tRNA processing enzymes. Central to these tRNA modifications is the precise biological roles played by such changes or lack thereof. Once these roles are defined, strategies eventually will emerge to develop therapeutics directed towards correcting hypomodified tRNA or modulation of the expression of tRNA modification enzymes. In this context, recent advances in the field of tRNA biology, detection methods of modified tRNA, advances in genomics and proteomics together with studies on animal models are promising for a better understanding of the complex mechanisms in tRNA modifications leading to neuronal disorders and/or neuroprotection. Such advances will stimulate research in novel tRNA modification based therapeutics.

## Author Contributions

AB, AC and NK conceived the content, AB and NK provided the ideas, supervised the work and wrote the critical review. MH, ID, TVC, AE and AC contributed to the content. All authors read and approved the final version of the manuscript.

## Conflict of Interest Statement

The authors declare that the research was conducted in the absence of any commercial or financial relationships that could be construed as a potential conflict of interest.

## Footnotes

^1^http://mods.rna.albany.edu/mods/

^2^http://modomics.genesilico.pl/
